# The Prevalence of Hyperthyroidism Amongst Atrial Fibrillation Patients in the National Guard Hospital, Riyadh, Saudi Arabia: A Cross-Sectional Retrospective Study

**DOI:** 10.7759/cureus.46791

**Published:** 2023-10-10

**Authors:** Mohammed K Bakarmom, Ali N ALmuhanna, Saleh A Alhussaini, Anas Alghamdi, Ihab Suliman

**Affiliations:** 1 School of Medicine, King Saud Bin Abdulaziz University for Health Sciences College of Medicine, Riyadh, SAU; 2 Cardiology, King Abdulaziz Medical City, King Abdulaziz Cardiac Center, Ministry of National Guard Health Affairs, Riyadh, SAU

**Keywords:** subclinical hyperthyroidism, risk factors, prevalence, atrial fibrillation, hyperthyroidism

## Abstract

Introduction

The thyroid gland is the largest pure endocrine gland. It is also responsible for the production of multiple important hormones that regulate heart function. Dysfunction of the thyroid gland could lead to hyperthyroidism, which in turn leads to many lipid and cardiovascular problems. One such cardiac condition that is highly associated with hyperthyroidism is atrial fibrillation. Atrial fibrillation is when abnormal electrical impulses suddenly start firing in the atria. With a prevalence of 16% to 60% of atrial fibrillation in hyperthyroid patients. To the best of our knowledge, no similar study was conducted in Saudi Arabia.

Objectives

Our study aims to investigate the percentage of people with atrial fibrillation that is caused by hyperthyroidism in the National Guard Hospital. Moreover, we hope that this study will improve the quality of life of these affected patients and add to their knowledge of medicine.

Methods

Since the population size has increased by approximately 4000 patients in the last five years, we estimated the sample size based on a confidence interval of 95% and a level of significance of 5%, which is 350 patients. We included all patients with atrial fibrillation due to hyperthyroidism in KAMC-R and patients’ records from 2015 to 2020. Also, we excluded all patients with atrial fibrillation due to other causes. In addition, the sampling method we used was convenient sampling.

Result

Out of 1100 patients with hyperthyroidism or subclinical hyperthyroidism, 40 or 3.6% of the patients had atrial fibrillation, while the rest were distributed among dilated cardiomyopathy, diabetes mellitus, heart failure, and other risk factors for atrial fibrillation.

Conclusion

We conclude that hyperthyroidism is a risk factor for atrial fibrillation; 3.6% of hyperthyroidism patients have atrial fibrillation. Most of the patients are elderly, and more than half (58%) are female.

## Introduction

The thyroid gland is the largest pure endocrine gland, and it is located anterolaterally to the thyroid cartilage in the neck. One of its functions is to produce two major hormones, which are thyroxine (T4) and triiodothyronine (T3). Between these two hormones, T3 is more biologically active. Moreover, T4 will be converted to T3 as it enters the target cell through deiodinase enzymes. These hormones play a main role in different metabolic pathways, such as cardiac functions and lipoprotein lipase [1]. The well-known effect of thyroid hormones on heart function is hyperdynamic circulation, which is characterized by increased cardiac contractility, heart rate, increased preload, and decreased systemic vascular resistance (SVR), which can result in increased cardiac output [2].

In normal physiology, one of the functions of the pituitary gland is to control T3 and T4 levels through a feedback mechanism. When the levels of T3 and T4 are decreased, the pituitary gland begins to produce TSH to excite the thyroid gland to produce its hormones. However, in hyperthyroidism, the pituitary gland production of thyroid-stimulating hormone (TSH) will be decreased, thus leading to an increase in the thyroid hormones (T3) and (T4). In addition, hyperthyroidism, thyroid overactivity, or thyrotoxicosis is when the thyroid gland becomes unusually active and secretes hormones, mainly thyroxine (T4), more than usual. Hyperthyroidism is a common disease affecting approximately 2-5% of women who are in their second and fourth decades of life. There are three main diseases or disorders that usually account for the main causes of hyperthyroidism: Graves’ disease, which is the main cause, accounts for 80% of cases; Plummer disease, or multinodular toxic goiter, accounts for 15%; and finally, toxic thyroid adenoma accounts for 2% of cases [3]. Moreover, there are many causes of hyperthyroidism, such as thyroiditis, thyroiditis factitial, some drugs such as amiodarone, and some cancers and tumors as well [4]. However, these causes rarely cause hyperthyroidism in comparison to the first three that were mentioned. "In addition, hyperthyroidism leads to many symptoms, one of which is atrial fibrillation, with a prevalence of 16 to 60% of atrial fibrillation in hyperthyroid patients [5]. Also, atrial fibrillation is the most common cardiac arrhythmia; 1% to 2% of the overall population is affected. It is characterized by fast and disorganized atrial activation leading to failure of the atrial function, which can be diagnosed on an ECG by the disappearance of P-waves and irregular QRS complexes. There are many causes of atrial fibrillation, some of which are cardiac diseases like myocardial infarction and heart failure, age, hyperthyroidism, stress, smoking, obesity, and high blood pressure. The most dangerous consequences of atrial fibrillation are thromboembolism and stroke; thus, anticoagulant medications must be prescribed to every atrial fibrillation patient.

The paper published by the American Heart Journal titled “Subclinical hyperthyroidism as a risk factor for atrial fibrillation” is a similar research issued on November 5, 2001. They studied 23,638 people who were classified based on the concentration of their serum thyrotropin, which is a hormone that stimulates the release of TSH and prolactin, into three groups. They concluded that "a low serum thyrotropin concentration is associated with a less than fivefold higher likelihood of the presence of atrial fibrillation, with no significant difference between subclinical and overt hyperthyroidism [6]."

To the best of our knowledge, no similar study was conducted in Saudi Arabia. Therefore, the aim of this research is to estimate the percentage of people with atrial fibrillation that is caused by hyperthyroidism in the National Guard Hospital. Moreover, we hope that this study will improve the quality of life of these affected patients and add to their knowledge of medicine.

## Materials and methods

This study is a cross-sectional study conducted in one of the largest tertiary care centers in Saudi Arabia, which is King Abdulaziz Medical City in Riyadh (KAMC-R). Since the population size has increased by approximately 4000 patients in the last five years, we estimated the sample size based on a confidence interval of 95% and a level of significance of 5%, which is 350 patients. We included all patients with atrial fibrillation due to hyperthyroidism in KAMC-R and patients’ records from 2013 to 2020. Also, we excluded all patients with atrial fibrillation due to other causes. In addition, the sampling method we used was convenient sampling.

We retrieved data from the medical records of all patients with hyperthyroidism to evaluate the risk of atrial fibrillation in these patients. We sent a request to medical records in KAMC-R to obtain the data of all patients who met the criteria. We made a well-structured data collection sheet that includes hyperthyroidism-related labs, tests, and whether the patient has atrial fibrillation or not. Moreover, we included age, gender, and family history as hyperthyroidism risk factors. Removing any type of data that identifies the patients, including names, phone numbers, and IDs, made sure that any necessary identifying information was replaced by a serial number.

For the data management and analysis, we used Microsoft Excel (Microsoft® Corp., Redmond, WA) for data entry, while JMP used it for data analysis. Categorical data are presented as frequencies and percentages (n; %), such as gender and education level, while numerical data such as age and overall perception score are presented as mean and standard deviation. The chi-square test was used to assess the association between the level of perception (low, medium, and high) and other baseline characteristics such as gender and education level. Finally, a test will be considered significant if the p-value is <0.05.

## Results

Through an extensive retrospective chart review of 1100 patients, we gathered valuable insights into the prevalence and characteristics of atrial fibrillation. Among the studied patients, 40 individuals were diagnosed with either hyperthyroidism or subclinical hyperthyroidism, with atrial fibrillation. The patients also exhibited a diverse range of risk factors, including dilated and ischemic cardiomyopathy, diabetes mellitus, hypertension, heart failure, chronic ischemic heart disease, pneumonia, and other comorbidities linked to atrial fibrillation.

Our study further shed light on the gender distribution among patients with atrial fibrillation and hyperthyroidism, or subclinical hyperthyroidism. Notably, approximately 58% of these patients were female, while the remaining 43% were male. This observation emphasizes the potential gender-related susceptibility to the combined occurrence of atrial fibrillation and hyperthyroidism.

**Figure 1:**
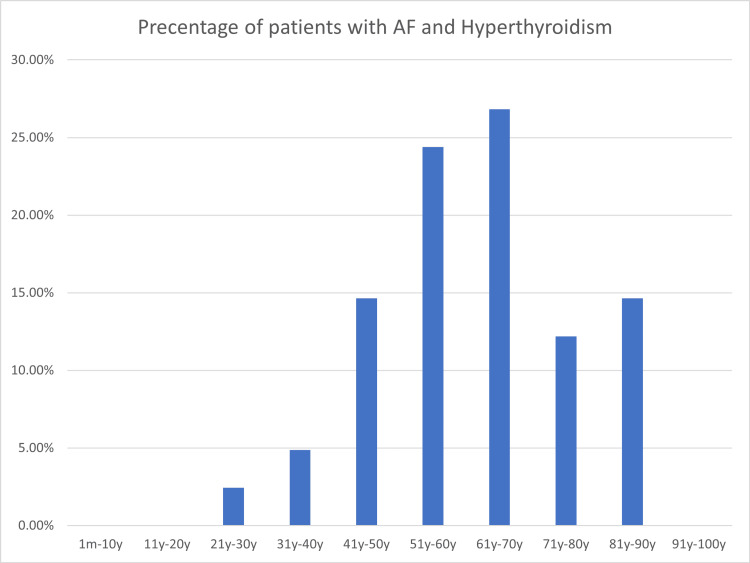
Age of patients with atrial fibrillation and hyperthyroidism

Regarding the age demographics, our findings revealed that the majority of patients presenting with atrial fibrillation and hyperthyroidism or subclinical hyperthyroidism were adults aged 41 years or older, accounting for 95% of the cases. Among this age group, 27.5% of patients fell within the 51-60-year range, while an equal percentage belonged to the 61-70-year age bracket. The mean age of presentation for these patients was determined to be 62 years, with a minimum age of 21 years and a maximum age of 90 years. The age distribution displayed a standard deviation of 15 years (Figure 1), reflecting the considerable variation in the age of onset among individuals with atrial fibrillation and hyperthyroidism or subclinical hyperthyroidism.

## Discussion

The association between atrial fibrillation and hyperthyroidism is known. However, the local data on this issue are limited and dated. The most common risk factors for atrial fibrillation, such as hyperthyroidism, hypertension, and cardiomyopathy, were established through our study of the matter.

Our comprehensive study suggests that both overt and subclinical hyperthyroidism elevate the risk of developing atrial fibrillation, far surpassing the anticipated levels, as clearly demonstrated in the extensive research we examined [6]. Additionally, we observed that several other prevalent risk factors, such as diabetes mellitus, hypertension, cardiomyopathy, ischemic heart disease, heart failure, and pneumonia, were consistently associated with atrial fibrillation, aligning perfectly with the findings reported in the referenced study [6]. Furthermore, our investigation revealed that advanced age serves as a prominent risk factor for atrial fibrillation, as affirmed by the results. It was evident that the older the patients became, the higher their susceptibility to both atrial fibrillation and hyperthyroidism, which strongly corroborates the discussions presented in the study by Zammitt and O'Brien [6]. In addition, we found that the most common age for the patients to develop atrial fibrillation and hyperthyroidism was from the age of 40 until the age of 70, with a drop in the number of patients between the ages of 71 and 80, and then it shows a significant increase that happens in patients between the ages of 89 and 90, and it is worth noting that the number of patients is the lowest at the ages of 21-30. However, it is important to note that our study deviates from the mentioned research [6] concerning the influence of gender on the incidence of atrial fibrillation. Contrary to their findings, our study uncovered a noteworthy distinction, indicating that females exhibit a greater susceptibility to hyperthyroidism and subclinical hyperthyroidism, consequently elevating their risk of developing atrial fibrillation.

The strengths of our study include our relatively large sample size, and the detailed documentation of the relationship between atrial fibrillation and hyperthyroidism/subclinical hyperthyroidism, and other atrial fibrillation comorbidities.

## Conclusions

We conclude from our comprehensive analysis that hyperthyroidism poses a risk factor for the development of atrial fibrillation. This condition is observed in approximately 3.6% of patients diagnosed with atrial fibrillation, highlighting the association between the two conditions. In addition to hyperthyroidism, several other risk factors contribute to the onset of atrial fibrillation. These include dilated and ischemic cardiomyopathy, diabetes mellitus, hypertension, heart failure, and pneumonia. It is crucial to consider these factors when assessing the overall risk profile of individuals susceptible to atrial fibrillation.

In our research, we found that most of the patients with atrial fibrillation and hyperthyroidism are in the elderly demographic, with ages ranging between 50 and 70 years old, as they are more prone to developing atrial fibrillation. Furthermore, it is worth noting that a majority of the patients affected by this condition were female, accounting for over half (58%) of the total cases analyzed. However, it is important to acknowledge the limitations of our study, which was conducted exclusively at the National Guard Hospital in Riyadh, Saudi Arabia. To obtain a more comprehensive understanding of atrial fibrillation and its association with hyperthyroidism, further investigations encompassing larger patient cohorts from diverse geographical regions are warranted. The conduct of additional studies will facilitate a more accurate estimation of the prevalence of atrial fibrillation and its relationship with hyperthyroidism on a global scale.
